# 5-FU-induced cardiac toxicity - an underestimated problem in radiooncology?

**DOI:** 10.1186/1748-717X-7-212

**Published:** 2012-12-15

**Authors:** Felix Steger, Matthias G Hautmann, Oliver Kölbl

**Affiliations:** 1 Department of radiotherapy, University of Regensburg, Franz-Josef-Strauss-Allee 11, Regensburg, 93053, Germany

## Abstract

**Background:**

5-Fluorouracil (5-FU) is an antimetabolite, which is frequently used as chemotherapeutic agent for combined chemoradiotherapy. The purpose of this study was to present the clinical course of three patients who developed severe cardiac toxicity by 5-FU and to give a review of the literature on the cardiotoxic potential of 5-FU.

**Results:**

Cardiotoxicity is a rare, but relevant side effect of fluoropyrimidines. It comprehends a wide spectrum of side effects, from electrocardiogram changes (69% of cardiac events) to myocardial infarction (22%) and cardiogenic shock (1%).

In this case series three patients with cardiotoxic events during chemoradiotherapy including 5-FU, the reaction's characteristics and their influence on further therapy are described. Two of the patients could not be treated with 5-FU any more because they had developed a myocardial ischemia, which was most likely caused by fluorouracil. Another patient, who complained about typical angina pectoris during 5-FU-infusion and had a new left anterior hemiblock, was reexposed with prophylactic administration of nitrendipine.

**Conclusion:**

Cardiotoxicity caused by 5-FU is an underestimated problem in radiooncology. Especially patients without history of cardiac disease are often treated as out-patients and therefore without cardiac monitoring. Consequently asymptomatic and symptomatic cardiac events may be overlooked. The benefit of prophylactic agents remains unclear, so close cardiac monitoring is the most established method to prevent manifest cardiotoxic events.

## Background

5-Fluorouracil (5-FU) is an antimetabolite, which, in radiooncology, is frequently used as chemotherapeutic agent during simultaneous chemoradiotherapy. It is mainly applied in neoadjuvant and definitive chemoradiotherapy of gastrointestinal malignancies and carcinoma of head and neck. Fluoropyrimidine therapy may cause frequent side effects like myelosuppression, mucositis, nausea, emesis and hand-foot-syndrome, but is also associated with toxic cardiac reactions [1,2]. These comprehend a wide spectrum from asymptomatic electrocardiogram (ECG)-changes and subacute, transient arrhythmias through to potentially life threatening events like myocardial ischemia and cardiogenic shock [3,4]. If one considers the frequency of 5-FU-induced cardiac side effects as documented in the literature, cardiac toxicity represents a problem which is underestimated in radiooncology. Those patients who do not have cardiovascular risk factors are often treated as outpatients and without cardiac monitoring. Consequently asymptomatic as well as symptomatic cardiac events remain undiscovered. This leads to a discrepancy between reported and real toxicity, concerning both asymptomatic ECG-changes [5] and subacute, but symptomatic incidents which could not be observed or do not come up in the conversation between patient and physician.

A meta-analysis by Saif et al. shows the distribution and frequency of cardiac reactions. Hence pectoral angina, myocardial infarction and arrhythmias belong to the most common cardiac events caused by fluoropyrimidines. The patients who are taken into account in this analysis were treated for gastrointestinal malignancies, cancer of head and neck as well as mamma carcinoma without concurrent radiotherapy. 14% of them had a history of cardiac disease, cardiac risk factors were known with 37% of the patients. Also in the normal population, the prevalence of cardiovascular disease is high. According to data of the American Heart Association in the USA above 61 million people suffer from cardiovascular disease.

The following data clearly demonstrate the relevance of the cardiotoxic reactions during 5-FU therapy: 8% of the persons with cardiac events died after primary exposition – after being reexposed, the rate of lethal complications reached 13% [6].

Despite cardiotoxicity of fluorouracil is a rare event, it can be regularly seen. Three cases of cardiotoxicity during chemoradiotherapy containing 5-FU-infusion are described below.

## Case presentation

### Case 1

A 73-year-old man was treated for an esophageal carcinoma. The diagnosis of carcinoma was made when the patient developed a symptomatic, but subacute non-ST-segment elevation myocardial infarction (NSTEMI). Basic diagnostic showed an advanced anaemia with hemoglobin concentration of 5.9 g/dl. Further investigation could explain this by a distal adenocarcinoma of the esophagus (cT3 cN1 cM0 G2). Coronary angiography provided a medium grade stenosis both of the anterior descendant branch of left coronary artery and right coronary artery without any need for intervention. Echocardiographically an ejection fraction of 60% and a low grade diastolic dysfunction could be demonstrated. Given the moderate findings of diagnostics, the myocardial infarction was seen in connection with advanced anaemia. Hence, in interdisciplinary consensus, primary chemoradiotherapy with continuous infusion of 5-FU (1000 mg/qm per day, day 1 – 4) and cisplatinum (60 mg/qm, day 1) including cardiac monitoring was indicated. The therapeutic concept included irradiation of distal esophagus including locoregional lymphatic region with single doses of 1.8 Gy to a total dose of 66.6 Gy. Before the beginning of the therapy the patient's medication was adapted to the new diagnosis of coronary artery disease and myocardial infarction.

**Figure 1:**
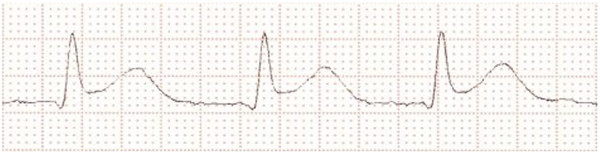
**ECG after beginning of pectoral angina.** New significant ST-segment elevation.

During the first cycle of concurrent chemotherapy, after approximately 93 hours of 5-FU-infusion (total applicated dose about 6800 mg), the patient developed an angina pectoris, so that application had to be stopped. Symptoms completely disappeared within few minutes for four hours, then the patient again complained about retrosternal chest pain and ECG showed new tachycardia. The patient was transferred to intensive care unit for monitoring, and further diagnostics revealed new significant ST-segment elevations (Figure 1), negative heart enzymes and intermittent atrial fibrillation. Consequently, the patient was treated with digitoxin, the dose of bisoprolol was increased and therapeutic anticoagulation was carried out. In the course of the day, ST-segment changes as well as atrial fibrillation disappeared and the patient became asymptomatic. Few ventricular extrasystoles could be detected in long-term ECG.

Taking all into conclusion, findings were interpreted as 5-FU-induced transient myocardial ischemia with synchronal atrial fibrillation. Because of the increased risk of recurrent cardiotoxicity, no further chemotherapy was applied. Radiotherapy could be completed without further complications.

### Case 2

A 49-year-old patient had a rectal carcinoma (uT3 uN1 cM0, adenocarcinoma G2, ranging from the anocutaneous line to 8 cm above) and was treated with neoadjuvant chemoradiotherapy including continuous 5-FU-infusion (1000 mg/qm per day, day 1 – 5) in first and fifth weeks of therapy. Radiotherapy was carried out with single doses of 1.8 Gy five times a week to a total dose of 50.4 Gy. At the time of diagnosis, the male patient was in good general constitution with no history of cardiac disease. He declared a nicotine abuse of 15 cigarettes per day, furthermore his father had died of myocardial infarction at the age of 63 years. Pretherapeutic ECG did not show any signs of myocardial ischemia, infarction or arrythmia.

During the application of the first cycle of 5-FU the patient developed a typical angina pectoris approximately 54 hours after the beginning of infusion. Symptoms improved after treatment with nitroglycerin and cessation of 5-FU-application (total applicated dose about 4400 mg). The ECG performed immediately afterwards revealed a new left anterior hemiblock, above that the following day exercise electrocardiography showed T-negativations in aVL and V2 without clinical symptoms. However, there were no signs of ischemia in myocardial scintigraphy even during exercise, furthermore no hypokinesia could be found echocardiographically.

Taking these diagnostic results into consideration and in the absence of clinical symptoms, it was decided to continue simultaneous 5-FU-therapy with prophylactic administration of a calcium channel blocker. A second cycle of chemotherapy was given with concurrent taking of nitrendipine 10 mg two times a day. In these conditions neoadjuvant chemoradiotherapy could be continued without further complications.

### Case 3

A 59-year-old patient who had an adenocarcinoma of the rectum (uT2 cN1 cM0 G2, 6–10 cm from the anocutaneous line), was treated with neoadjuvant chemoradiotherapy, too. The male patient was in good general constitution, moreover no pre-existing cardiac disease or risk factors apart from obesity could be determined. Consequently, neoadjuvant chemoradiotherapy with continuous 5-FU-infusion (1000 mg/qm per day, day 1 – 5) was indicated. Radiotherapy was applied by analogy to the concept of the second patient.

During the first cycle the patient developed pectoral angina approximately 65 hours after the beginning of infusion. Consequently, application of 5-FU was stopped. ECG did not show any signs of ischemia, but symptoms were responsive to nitroglycerin. Six hours after onset of the symptoms a significant elevation of troponin I could be observed, so that a NSTEMI was diagnosed. Coronary angiography, which was consequently performed, did not reveal any relevant stenosis. Thus, the described cardiac event was interpreted as 5-FU-induced myocardial ischemia.

As a consequence, it was decided not to reexpose the patient with fluorouracil and the further neoadjuvant therapy was carried out as radiotherapy alone.

## Conclusions

In addition to the cardiac side effects mentioned above there are further cardiotoxic reactions described in the literature. The review by Saif et al. specifies the following events and their distribution in relation to the total number of cardiac side effects [6]: ST-T changes (69% of cardiac events), Angina pectoris (45%), Arrhythmias (23%), Myocardial infarction (22%), Abnormal cardiac enzymes (12%), Pulmonary embolism (5%), Cardiac arrest/pericarditis (1.4%).

As regards to general frequency of cardiotoxic side effects there are inconsistent data in the literature, but for the most part the risk is considered to be a single-digit percentage. It ranges from 1.6% of 5-FU-exposed patients [4] through 7.6% [7] in collectives that are not preselected involving patients having a history of cardiac disease, to 8% [8] within a group of heart-healthy patients. Due to different inclusion criteria, this data is only comparable to a limited extent. Above that some studies showed an influence of 5-FU-application-protocol to frequency of cardiotoxic side effects. If fluorouracil is applied as continuous infusion, the risk of cardiotoxicity seems to be higher than with bolus application or short infusion [9,10].

Although some influencing factors are well known, the mechanism which is responsible for the effects mentioned above remains poorly defined. The following causes are mainly discussed in the literature: direct myocardial damage, vasospasm, increased oxygen demand, rheological effects, autoimmune reaction, cytotoxic effects, endothelial changes and impurities [6,11,12]. It remains unclear which of the effects mentioned above is responsible for cardiotoxicity of fluoropyrimidines. The results to date do not lead to a distinct conclusion. Interestingly a connection between circulating plasma levels of fluorouracil and cardiac side effects does not seem to exist [13]. Furthermore, patients with an insufficiency of dihydropyrimidindehydrogenase (DPD), the key enzyme of fluoropyrimidine metabolism, do not show elevated rates of cardiotoxic reactions [14].

If there is a cardiotoxic event during primary application of 5-FU, the question arises if there are viable prophylactic options. Due to the theory of vasospasm-induced myocardial ischemia or angina pectoris, calcium channel blockers and nitrates are primarily used, similar to the treatment of variant angina. In the second reported case nitrendipine was applied successfully to enable a rechallenge. We opted for this treatment due to the fact that the patient had no history of cardiac disease and had not developed an apparent myocardial infarction during the first cycle of concurrent chemotherapy. Under such conditions, therapy could be continued without further complications. In case of the other patients the chemotherapy was discontinued because the rechallenge to fluorouracil is associated with an increased risk for serious complications. The first patient had shown a new ST-segment elevation and previously had suffered a myocardial infarction, the third patient developed a NSTEMI which, in all likelihood, was 5-FU-induced, considering that coronary angiography did not show any relevant stenosis.

As regards the prophylactic use of calcium channel blockers and nitrates, there are inconsistent data in previous studies. Cianci et al. treated two patients after first cardiotoxic event with transdermal nitrates, above that the dose of fluorouracil was reduced by 10-20%. During rechallenge no further cardiotoxic event appeared [15]. There are further smaller publications which describe the effectiveness of administration of prophylactic drugs. On the other hand, some other studies could not confirm the effect of calcium channel blockers or nitrates in reducing the risk of cardiotoxicity. In a collective of seven patients cardiotoxicity could not successfully be prevented [16]. Eskilsson and Albertsson compared within a total number of 97 patients a group without prophylaxis with patients who received verapamil (120 mg three times a day), and their analysis did not demonstrate a significant difference between these groups with 12% vs. 13% recurrent cardiotoxicity [17]. However, at the moment there are no prospective randomised studies that examine this issue.

Despite from that, administration of prophylactic agents is widespread, all the more so as there are only few alternatives for 5-FU in simultaneous chemoradiotherapy. According to present data, also Capecitabine, the oral prodrug of fluorouracil, has a comparable risk of cardiotoxicity [18,19]. What is more, there are no chemotherapeutic options with good evidence for effectiveness and favourable risk profile, at least as far as gastrointestinal malignancies are concerned.

Moreover, few data are available on predictive markers for cardiac side effects of fluoropyrimidines. Elevated plasma level of brain natriuretic peptide (BNP) is significantly correlated with increased risk of cardiotoxicity during 5-FU-infusion [20], but data still need to be validated.

The most reliable method for early detection of cardiac events during chemotherapy with 5-FU continues to be close cardiac ECG-monitoring. Hereby myocardial ischemia or arrhythmias can be detected before becoming clinically manifest. However, to achieve that, close monitoring of both patient and ECG is necessary.

In summary, cardiotoxicity of 5-FU is an underestimated problem in radiooncology. There are no reliable chemotherapeutic alternatives especially for therapy of gastrointestinal malignancies. Moreover, the role of predictive markers still needs to be defined exactly. Prophylactic application of calcium channel blockers or nitrates is an option to enable rechallenge with 5-FU after cardiotoxic reaction during primary therapy, but more evidence for effectiveness of these drugs in preventing cardiotoxic reactions is needed. In conclusion, close cardiac monitoring with, if necessary, cessation of 5-FU-infusion continues to be the most established method.

### Consent

Written informed consent was obtained from the patients for publication of this report. A copy of the written consent is available for review by the Editor-in-Chief of this journal.

## Abbreviations

5-FU: 5-Fluorouracil; ECG: Electrocardiogram; NSTEMI: Non-ST-segment elevation myocardial infarction; BNP: Brain natriuretic peptide.

## Competing interests

The authors declare that they have no competing interests.

## Authors’ contributions

FS identified the patients, registered the patients' data and drafted the manuscript. MGH contributed to analysis of data and reviewed the manuscript. OK was revising the manuscript critically. All authors read and approved the final manuscript.
